# Revitalizing Colchicine: Novel Delivery Platforms and Derivatives to Expand Its Therapeutic Potential

**DOI:** 10.3390/ijms26157591

**Published:** 2025-08-06

**Authors:** Natallia V. Dubashynskaya, Anton N. Bokatyi, Mikhail M. Galagudza, Yury A. Skorik

**Affiliations:** 1 Branch of Petersburg Nuclear Physics Institute Named by B.P. Konstantinov of National Research Centre «Kurchatov Institute»—Institute of Macromolecular Compounds, Bolshoi pr. VO, 31, St. Petersburg 199004, Russia; 2 Almazov National Medical Research Centre, Akkuratova str., 2, St. Petersburg 197341, Russia

**Keywords:** colchicine, drug delivery systems, prodrug, codrug, immune-mediated inflammatory diseases

## Abstract

Colchicine is a potent alkaloid with well-established anti-inflammatory properties. It shows significant promise in treating classic immune-mediated inflammatory diseases, as well as associated cardiovascular diseases, including atherosclerosis. However, its clinical use is limited by a narrow therapeutic window, dose-limiting systemic toxicity, variable bioavailability, and clinically significant drug–drug interactions, partly mediated by modulation of P-glycoprotein and cytochrome P450 3A4 metabolism. This review explores advanced delivery strategies designed to overcome these limitations. We critically evaluate lipid-based systems, such as solid lipid nanoparticles, liposomes, transferosomes, ethosomes, and cubosomes; polymer-based nanoparticles; microneedles; and implants, including drug-eluting stents. These systems ensure targeted delivery, improve pharmacokinetics, and reduce toxicity. Additionally, we discuss chemical derivatization approaches, such as prodrugs, codrugs, and strategic ring modifications (A-, B-, and C-rings), aimed at optimizing both the efficacy and safety profile of colchicine. Combinatorial nanoformulations that enable the co-delivery of colchicine with synergistic agents, such as glucocorticoids and statins, as well as theranostic platforms that integrate therapeutic and diagnostic functions, are also considered. These innovative delivery systems and derivatives have the potential to transform colchicine therapy by broadening its clinical applications while minimizing adverse effects. Future challenges include scalable manufacturing, long-term safety validation, and the translation of research into clinical practice.

## 1. Introduction

The global burden of immune-mediated inflammatory diseases (IMIDs)—encompassing diseases of the respiratory system, joints, skin, gastrointestinal tract, and central nervous system—continues to rise, representing a major challenge to healthcare systems due to their severity, chronicity, and association with substantial morbidity and mortality [1,2]. The family of IMIDs is remarkably heterogeneous, with systemic autoimmune diseases (e.g., rheumatoid arthritis and systemic lupus erythematosus) on one side and chronic hypersensitivity disorders (e.g., T2-high bronchial asthma) on the other. Generally, these disorders are characterized by a dysregulated immune response, leading to chronic inflammation in multiple areas that are simultaneously or sequentially affected, overproduction of cytokines, and progressive organ damage [3]. The pathogenesis of IMIDs involves a complex interplay between genetic susceptibility, driven by multiple common genetic variants, and environmental triggers (or risk factors) involving periodontal disease, intestinal dysbiosis, infectious disease, smoking, and obesity [4]. IMIDs not only cause inflammation-mediated tissue/organ dysfunction in specific location(s), but also increase the risk of cardiovascular and metabolic diseases, bone abnormalities, and cognitive impairment, all of which further adversely affect the quality of life and the overall prognosis of the patients [2]. In particular, inflammation with associated elevation of proinflammatory mediators in the circulation is recognized as an important contributor to endothelial dysfunction, later culminating in the formation of atherosclerotic plaque [5]. Atherosclerosis is currently the underlying cause of nearly 50% of deaths worldwide, mainly due to severe ischemic injury of the brain and/or heart [6,7]. Current IMID management relies on diverse anti-inflammatory agents, including non-steroidal anti-inflammatory drugs, glucocorticoids, immunosuppressants, biological drugs, and antibody–drug conjugates [8,9]. While effectively inducing remission, conventional therapies, such as corticosteroids, immunosuppressants, and biological therapies, often induce broad immune suppression, thereby elevating the risks of serious, sometimes generalized, infections and malignancies [10]. This underscores an unmet need for therapeutic strategies capable of selectively targeting pathological immune pathways while preserving overall immune competence. One reasonable solution might come from the chemical modification and/or targeted delivery of natural molecules that have a long history of use in medicine, as exemplified in this review.

Colchicine (COL), a tricyclic alkaloid derived from *Colchicum autumnale* L. with over two millennia of documented medicinal use [11], holds a unique position among anti-inflammatory agents. Approved in 2009 by the FDA for treating gout and familial Mediterranean fever [12,13], its therapeutic relevance has expanded significantly. Elucidation of the pivotal role of inflammation in atherosclerosis has fueled interest in the potential use of COL for plaque stabilization or even regression. Recent research demonstrates its efficacy in preventing atherosclerotic plaque growth and destabilization via inhibition of intimal inflammation, oxidative injury, platelet aggregation, and modulation of autophagy [14]. Deciphering the molecular mechanisms, particularly suppression of autoinflammatory pathways in IMID immunopathogenesis [15,16,17], resulted in the landmark 2023 FDA approval of low-dose COL (0.5 mg, LODOCO^®^) for treatment of atherosclerosis with proven clinical benefit [18]. This evidence supports using COL as a valuable adjunct to established antiatherosclerotic therapies [19,20], marking a major therapeutic advance. The anti-tubulin mechanism of action of COL disrupts mitotic progression by arresting cells in metaphase, thereby inducing apoptosis. Additionally, COL inhibits tumor angiogenesis by impairing the formation of the vascular network and destabilizing existing tumor vasculature. COL also suppresses mitochondrial metabolism in cancer cells. Together, these multimodal mechanisms support the classification of COL as a potent antitumor agent [21,22].

However, the repositioning of COL for chronic conditions like atherosclerosis is hampered by significant pharmacokinetic and safety limitations inherent to its conventional oral administration. These include extensive first-pass metabolism, highly variable bioavailability, dose-limiting gastrointestinal toxicity [11,23], and a critically narrow therapeutic index due to high cytotoxicity against normal cells [24,25]. Some of these factors have accounted for the disappointing results of the COLCOT (Colchicine Cardiovascular Outcomes Trial), which demonstrated a 23% reduction in the primary composite endpoint in patients with myocardial infarction in the COL vs. placebo group at the expense of a greater incidence of serious side effects such as pneumonia [26]. These constraints necessitate innovative strategies to enhance the therapeutic utility of COL. Advanced drug delivery systems, particularly those using nanotechnology, offer a promising solution by enabling optimized release profiles along with targeted delivery into the focus of inflammation, thereby improving pharmacokinetic properties, reducing toxicity, and potentially widening the therapeutic window.

Despite the growing clinical importance of COL and the clear need for improved delivery, comprehensive reviews focusing specifically on delivery systems for COL and its derivatives remain scarce. Lei et al. [11] highlighted various administration strategies emphasizing toxicity reduction and efficacy enhancement, identifying transdermal delivery as particularly promising and underscoring the need for expanded formulation development. Other reviews have concentrated on the chemical modification of COL to enhance efficacy or reduce toxicity. Gracheva et al. [27] detailed the synthesis, structure–activity relationships (SAR), and hybrid compounds based on COL alkaloids, while Ghawanmeh et al. [24] summarized recent synthetic approaches, biological activities, SAR studies, and toxicity profiles of derivatives. Rubicondo et al. [28] provided a comprehensive overview of COL derivatives and their nanoformulations specifically for oncology applications.

This review summarizes recent advancements in improving the pharmacokinetic profile of COL using nanotechnology-based delivery systems, such as lipid- and polymer-based nanoparticles, microneedles, and implants. Through a critical evaluation of these innovative formulations, we identify strategies to optimize therapeutic outcomes in inflammatory and IMIDs and related pathologies. Key approaches include broadening COL’s clinical applications via chemical derivatization (e.g., prodrugs and codrugs) and combinatorial nanoformulations for co-delivery with synergistic agents, such as glucocorticoids and statins. We envision that this comprehensive analysis will catalyze the development of safer, more effective COL delivery systems.

## 2. Features of Colchicine as an Active Pharmaceutical Substance

### 2.1. Chemical Structure and Biopharmaceutical Properties of Colchicine

**Figure 1:**
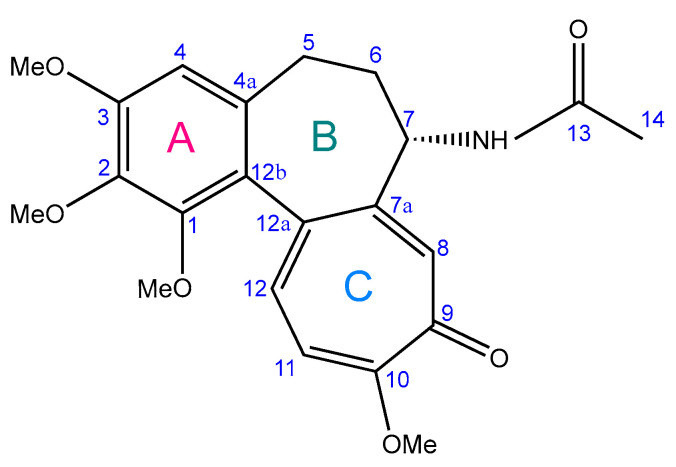
Chemical structure of colchicine (adapted from [28]).

COL is a tropolone-class alkaloid and the principal bioactive compound derived from *Colchicum autumnale* L., predominantly extracted from its seeds and roots [29,30]. Chemically designated as N-[(7S)-5,6,7,9-tetrahydro-1,2,3,10-tetramethoxy-9-oxobenzo[a]heptalen-7-yl] acetamide, it features a small, lipophilic structure comprising three distinct rings (Figure 1). Ring A is a methoxy-substituted benzene ring (methoxy groups at C-1, C-2, and C-3), while Ring C is a tropolone ring with a methoxy group at C-10. These rings mediate high-affinity binding of COL with tubulin, underpinning its pharmacological activity. Ring B, a seven-membered saturated ring bearing an acetamide group at C-7, contributes to the biophysical stability and thermodynamic properties of COL [27,28,31,32].

Following oral administration (typically at a dose of 1 mg), COL is rapidly absorbed in the gastrointestinal tract, achieving peak plasma concentration within approximately one hour [33]. However, its oral bioavailability exhibits considerable interindividual variability (24–88%) [34], and its large volume of distribution (~7 L/kg) reflects extensive tissue penetration. Approximately 40% of circulating COL binds to albumin. Despite an elimination half-life of 20–40 h due to enterohepatic recirculation, steady-state plasma concentrations after repeated 1 mg/day dosing remain low (0.3–2.5 ng/mL). Notably, the pharmacodynamic effects of COL correlate with its accumulation within leukocytes rather than with plasma levels [35,36]. This compound possesses a notoriously narrow therapeutic index, with fatalities reported following single doses as low as 7 mg [37,38].

COL undergoes significant hepatic metabolism, primarily via cytochrome P450 3A4 (CYP3A4)-mediated demethylation, while elimination occurs mainly through the gut (both the native drug and its metabolites) and, to a lesser extent (10–20%), via renal excretion. The efflux transporter P-glycoprotein (P-gp), expressed in intestinal, hepatic, and renal epithelia, plays a crucial protective role by actively transporting COL from the cell during drug excretion, thereby reducing COL toxicity [39,40]. This metabolic and excretory profile underlies significant drug–drug interactions. Concomitant use with potent P-gp inhibitors or CYP3A4 inhibitors markedly elevates plasma COL concentrations, increasing the risk of severe adverse effects [35,38]. This is particularly relevant given that several commonly used cardiovascular drugs, including certain calcium channel blockers and angiotensin II receptor blockers, possess an inhibiting effect on P-gp [41]. Furthermore, P-gp mediates the transport of cardiac glycosides and glucocorticoids (e.g., dexamethasone) [42,43], while CYP3A4 provides the metabolic conversion of statins, including atorvastatin and simvastatin [44,45], necessitating extreme caution during co-administration of these drugs with COL.

These challenging biopharmaceutical properties—variable absorption, extensive metabolism, P-gp/CYP3A4-dependent interactions, leukocyte-dependent pharmacodynamics, and a critically narrow therapeutic window—directly motivate the development of advanced delivery systems for COL. Such strategies as the chemical design of prodrugs, codrugs, conjugates, and nanoformulations aim to overcome these limitations and improve the absorption, distribution, metabolism, excretion, and toxicity (ADMET) profile of COL [46].

### 2.2. Colchicine Mechanism of Action

**Figure 2:**
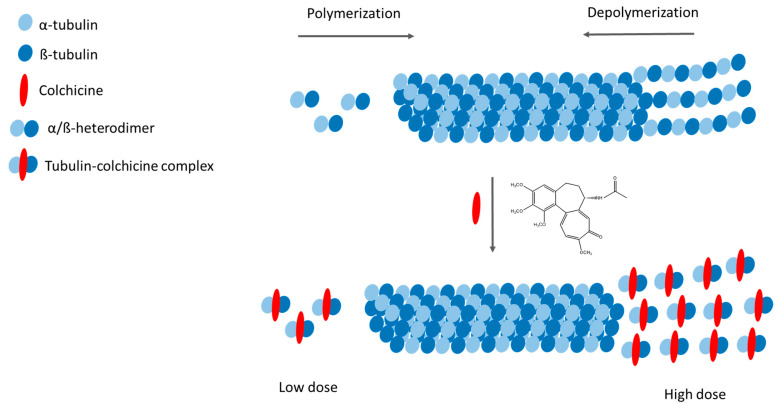
Mechanism of action of colchicine (adapted from [28]).

Despite extensive research, the mechanisms of anti-inflammatory action of COL remain not fully understood [13]. Its primary pharmacological target is tubulin, the structural protein of microtubules, which performs critical cellular functions, including chromosome segregation during cell division and the transport of cellular cargo [47]. COL binds with high affinity to α/β-tubulin heterodimers, inducing conformational changes that disrupt microtubule dynamics (Figure 2). This interaction exhibits dose-dependent effects, with low concentrations inhibiting microtubule assembly and higher concentrations promoting active depolymerization at the minus end. The resulting destabilization impairs mitotic spindle formation, leading to aberrant chromosome segregation and subsequent apoptosis [48].

These microtubule-disrupting properties underlie the dual pharmacological role of COL. On one hand, it demonstrates potent antineoplastic activity by inhibiting cancer cell migration, angiogenesis, and metastasis formation [49]. On the other hand, microtubule disruption triggers side effects across multiple cell types, particularly endothelial and immunocompetent cells, including monocytes, macrophages, and neutrophils. These include impaired cellular motility, reduced cytokine/chemokine synthesis, and suppressed adhesion molecule expression. Importantly, COL inhibits activation of caspase-1—the enzymatic core of the NLRP3 inflammasome—thereby blocking proteolytic maturation of two major pro-inflammatory cytokines: interleukin (IL)-1β and IL-18 [13]. As the master regulator of innate immunity, IL-1β stimulates production of downstream cytokines (IL-6, IL-8) and amplifies the inflammatory cascade [50]. Dysregulated NLRP3 activation drives both hereditary autoinflammatory syndromes and acquired conditions where endogenous crystals (e.g., urate and cholesterol in gout and atherosclerosis, respectively) serve as pathogenic triggers of inflammasome assembly [51,52,53].

Consequently, collagen-mediated inhibition of microtubule polymerization in immune cells results in the following: (1) suppression of multiple inflammatory cascades, (2) modulation of innate immune responses, and (3) other pleiotropic effects. Together, these mechanisms underlie the potent anti-inflammatory action of COL in IMIDs and related comorbidities, particularly in atherosclerosis. By suppressing NLRP3-mediated rapid IL-1β release, COL attenuates vascular inflammation—an effect substantiated by clinical trials demonstrating efficacy of low doses (0.5 mg/day) in patients with stable angina and myocardial infarction [13,54]. However, the interaction of COL with tubulin also mediates the systemic toxicity of the former, due to the impairment of essential cellular processes, including protein trafficking, endo/exocytosis, and mitotic progression. At the organ level, exemplified by the heart, this manifests as conduction abnormalities and contractile dysfunction. The cumulative effect is multi-organ dysfunction with potentially fatal outcomes [55], additionally threatened by certain nephrotoxicity risk arising from renal excretion of unmetabolized drug [56].

## 3. Colchicine Delivery Systems

**Figure 3:**
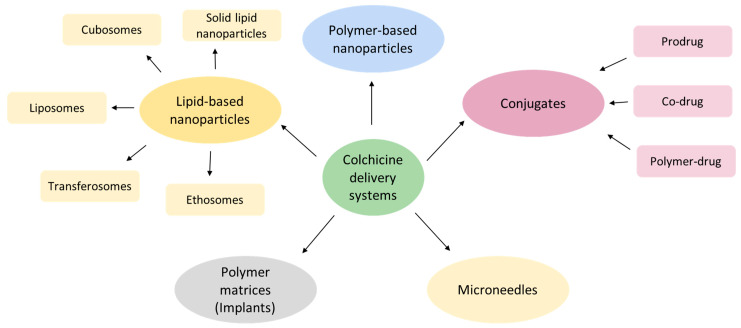
The diversity of colchicine delivery systems.

The clinical utility of COL remains limited by its narrow therapeutic index and off-target cytotoxicity, necessitating innovative delivery strategies to enhance safety and efficacy. A promising approach involves incorporating COL into advanced delivery platforms, including lipid- and polymer-based nanoparticles, hybrid systems, polymeric matrices, microneedles, and molecular conjugates, as illustrated in Figure 3. Nanotechnology-based delivery systems offer several advantages. In particular, they enable selective accumulation of the drug in the area of interest, provide controlled drug release kinetics, enhance drug stability, and prolong local residence time. Collectively, these benefits address critical ADMET limitations inherent to conventional COL administration [57].

By exploiting the enhanced permeability and retention (EPR) effect or active targeting, nanocarriers can preferentially deliver COL to inflamed tissues while minimizing exposure to healthy cells. This spatial control is especially relevant for IMIDs, as the effect of COL depends on its intracellular accumulation within infiltrating immune cells in the tissues. Furthermore, engineered release profiles decrease peak–trough plasma concentration fluctuations that contribute to dose-limiting toxicity, potentially widening the therapeutic window. Surface-modified nanoparticles also circumvent P-gp-mediated efflux in the gastrointestinal tract and liver, while polymeric encapsulation protects against premature CYP3A4 metabolism. Such systems thus represent a strategic solution to the pharmacokinetic vulnerabilities of the compound while amplifying its NLRP3 inflammasome-suppressing effects at target sites [57].

### 3.1. Lipid-Based Delivery Systems

Lipid-based delivery systems explore the favorable safety profiles and amphiphilic nature of phospholipids to produce versatile nanocarriers for optimizing COL delivery [30]. These lipid nanoparticles form structured vesicles with distinct hydrophilic and/or hydrophobic compartments capable of accommodating diverse payloads [58]. Encapsulation within lipid matrices enhances aqueous COL solubility and oral bioavailability while enabling controlled release kinetics and site-specific targeting—particularly when combined with surface ligands that recognize disease-specific biomarkers [59]. Representative systems include conventional liposomes, solid lipid nanoparticles, and deformable variants such as transferosomes, ethosomes, and transethosomes. This structural diversity allows for customization of multiple administration routes: systemic delivery via intravenous or oral pathways, and local approaches including transdermal and inhalational administration [60,61,62]. The compositional flexibility and design principles of these platforms are comprehensively detailed in prior work [30], underscoring their adaptability in overcoming biopharmaceutical challenges associated with COL.

#### 3.1.1. Solid Lipid Nanoparticles

Solid lipid nanoparticles (SLNs) represent a prominent class of lipid-based carriers engineered by dispersing solid lipids—typically stearic acid, glyceryl behenate, tripalmitin, cetyl palmitate, glyceryl monostearate, or tristearin—within an aqueous phase, followed by stabilization with surfactants, such as Poloxamer 188, Tween 20, or lecithins [63,64]. To optimize performance, liquid lipids like oleic acid or caprylic/capric triglycerides are often incorporated to inhibit lipid crystallization, enhance drug loading efficiency, and modulate release kinetics, yielding nanostructured lipid carriers with improved stability profiles [63,64]. Further surface modification, including coating with polyethylene glycol (PEG) and engraftment of targeting ligands, provides evasion of uptake by the cells of the reticuloendothelial system and specific retention in the inflamed tissue, respectively [64]. SLNs exhibit exceptional payload versatility, accommodating hydrophilic and lipophilic small molecules, macromolecules (proteins, polysaccharides), genetic material, vaccines, and antibodies [65]. Their nanoscale dimensions and lipophilic character facilitate efficient cellular uptake, penetration across physiological barriers (e.g., the blood–brain barrier), and the possibility of administration via parenteral, enteral, transdermal, and specialized routes [66,67,68,69].

For transdermal applications, SLNs enhance skin permeation due to their affinity for the cells of the stratum corneum, thereby providing sustained drug release. Incorporation into hydrogel matrices further prolongs local retention and maintains therapeutic concentrations [70]. Demonstrating this approach, Joshi et al. [71] developed SLNs using glyceryl monostearate and Tween 20 via ultrasonication, yielding particles with a diameter of 90–135 nm, with a low polydispersity index (PDI 0.2–0.3), and ζ-potentials of −24 to −18 mV. COL encapsulation efficiency ranged from 21 to 37%, with transdermal flux values of 4–9 μg/cm^2^/h and permeability coefficients of 7–18 cm/hr. Formulated into ethylcellulose-polyvinylpyrrolidone (PVP) (8:2) patches, these SLNs prolonged COL plasma exposure to 24 h and achieved 2.8-fold higher bioavailability versus patches containing free COL.

#### 3.1.2. Cubosomes

Cubosomes represent nanostructured liquid crystalline particles engineered from amphiphilic lipids, primarily unsaturated monoacylglycerols like glycerol monooleate and glycerol monolinoleate [72,73]. These lipids spontaneously self-assemble into four distinct mesophases—lamellar, reversed hexagonal, and bicontinuous cubic phases (Q230 and Q224)—with an exceptional capacity to maintain cubic phase integrity in aqueous environments, enabling biomedical applications [74]. Stabilization typically employs poly(ethylene oxide)-poly(propylene oxide) block copolymers such as Poloxamer 407 [74,75]. Their unique architecture features interwoven lipid bilayers and continuous water channels, providing an extensive surface area for the efficient encapsulation of diverse therapeutics: hydrophilic, hydrophobic, and amphiphilic compounds, along with macromolecules including proteins, peptides, and nucleic acids [76,77]. This structure further enhances drug permeation across epithelial barriers like skin and mucosa [78], with detailed structural characterization available in foundational work [72].

Demonstrating translational potential, Nasr et al. [74] developed glyceryl monooleate/Poloxamer 407 cubosomes for COL delivery, achieving particles with a diameter of 73 nm, +28 mV ζ-potential, and 32% encapsulation efficiency. Ex vivo permeation studies using rabbit abdominal skin revealed a permeability coefficient of 1.4 × 10^−2^ cm/h. Formulated within a 3% hydroxypropyl methylcellulose gel, in vivo evaluation in male Wistar rats demonstrated a 4.6-fold-higher COL bioavailability via transdermal application compared to an oral solution, validating cubosomes as a promising strategy for overcoming the pharmacokinetic limitations of COL.

#### 3.1.3. Liposomes

Liposomes represent versatile artificial nanovesicles constructed from natural or synthetic phospholipids, which use their inherent biocompatibility and membrane-mimetic properties for the efficient delivery of both hydrophilic and hydrophobic therapeutics [79,80]. Surface engineering further enhances their functionality through PEGylation for prolonged circulation, stimulus-responsive polymer coatings for triggered release, or ligand conjugation for tissue-specific targeting [81,82,83]. These modifications address the critical limitations of free anti-inflammatory drugs, such as poor bioavailability and systemic off-target effects, making liposomes an ideal carrier for COL.

Di Francesco et al. [84] exemplified this approach through liposomes co-encapsulating COL and methotrexate. Methotrexate was covalently conjugated to distearoyl phosphoethanolamine and embedded within the lipid bilayer, while COL occupied the aqueous core. The resulting vesicles (~100 nm, PDI 0.2, ζ-potential −40 mV) achieved a 30% encapsulation efficiency with sustained release profiles: 60% methotrexate and 70% COL released by 9 h, reaching completion at 24 h. In lipopolysaccharide-stimulated macrophages, these liposomes suppressed IL-1β and IL-6 expression by 3-fold while modulating atherosclerosis-relevant proteins—upregulating cholesterol-efflux transporter ABCA1 and downregulating receptors mediating lipid uptake (CD36 and SRA-1)—thereby attenuating oxidized low-density lipoprotein (LDL) accumulation and demonstrating therapeutic potential for vascular inflammation.

Similarly, Chennakesavulu et al. [62] engineered inhalable liposomes co-delivering COL and budesonide intended for the treatment of pulmonary fibrosis. Composed of dipalmitoylphosphoglycerol, hydrogenated soy phosphatidylcholine, and cholesterol, sub-100 nm vesicles achieved an exceptional 98% encapsulation efficiency and extended drug release over 24 h. In rats with bleomycin-induced lung fibrosis, pulmonary retention was prolonged while systemic exposure was reduced compared to free drugs.

For oncological applications, Chen et al. [85] developed tumor-targeted liposomes incorporating deuterated COL (reducing toxicity and extending plasma half-life time) coated with CD44-binding oligohyaluronic acid. The 120–130-nm particles (PDI 0.1, 70% encapsulation efficiency) exhibited pH-dependent release (20% at pH 7.4 vs. 60% at 5.0 over 24 h) and preferential tumor accumulation. In the 4T1 breast cancer model in mice, this platform reduced toxicity while enhancing antitumor efficacy, underscoring the synergy between deuteration and active targeting.

#### 3.1.4. Transferosomes

Transferosomes represent ultradeformable lipid vesicles engineered for enhanced transdermal delivery, combining phospholipids (e.g., soy or egg phosphatidylcholine derivatives) with edge activators—single-chain surfactants like sodium cholate, Tween variants, or dipotassium glycyrrhizinate [86]. These components confer exceptional stratum corneum permeability by destabilizing the vesicle bilayer, increasing flexibility and deformability beyond conventional liposomes, niosomes, or ethosomes [87,88,89]. Their penetration mechanism relies on high membrane elasticity and osmotic gradients at the application site [88]. Edge activators further modulate particle size and drug-loading capacity for both hydrophilic and lipophilic compounds, including macromolecules, while phospholipids enable bilayer formation for versatile encapsulation and modified release [90,91]. Additional surface functionalization with targeting ligands expands their utility [92,93].

El-Feky et al. [36] harnessed this platform for COL delivery, formulating transferosomes from soy lecithin and Tween 80. To optimize loading, COL was first complexed with β-cyclodextrin, achieving 42–94% encapsulation efficiency within vesicles of 70–138 nm in diameter and +16 to +23 mV ζ-potential. The system exhibited biphasic release, with 50% COL released within 1 h, followed by sustained release over 6 h. Ex vivo permeation studies using rat abdominal skin demonstrated 34.7% cumulative penetration of COL at 6 h. In monosodium urate/potassium oxonate-induced gout models in Sprague-Dawley rats, COL-loaded transferosomes significantly reduced paw edema and serum uric acid levels without skin irritation, validating their efficacy and safety for inflammatory disease management.

#### 3.1.5. Ethosomes

Ethosomes represent a specialized subclass of lipid vesicles characterized by a high ethanol content (20–45%), which significantly enhances skin permeability by fluidizing the stratum corneum lipid bilayers and increasing molecular mobility [94,95,96]. Composed of phospholipids with ethanol and terpenes as edge activators, ethosomes exhibit superior physicochemical stability, higher negative ζ-potentials, reduced particle sizes, and enhanced encapsulation efficiency compared to conventional liposomes, collectively enabling efficient transdermal delivery of macromolecules [97].

Zhang et al. [98] used this platform for COL by synthesizing borneol-conjugated dioleoylphosphoethanolamine ethosomes. The borneol modification reduced particle size while enhancing permeability parameters in vitro, yielding improved pharmacokinetics and reduced toxicity versus both free COL and non-modified vesicles. Complementary work by Yi et al. [99] demonstrated the permeabilization mechanism of borneol: disruption of stratum corneum alkyl chains and lipid extraction, particularly enhancing penetration of hydrophilic drugs (logP ≈ 0.5).

Transethosomes further optimize this approach by integrating transferosome and ethosome properties. Incorporating edge activators with high ethanol concentrations (≤30%), they exhibit irregular morphology, extreme deformability, and exceptional skin penetration capabilities [94,100]. Abdulbaqi et al. [101] formulated COL-loaded transethosomes using soybean phosphatidylcholine with Tween 20, sodium taurocholate, or Labrafil. Particles showed uniform sizes (80–150 nm), low PDI of 0.1–0.2, ζ-potentials ranging from −37 to +26 mV, and high encapsulation efficiency (65–85%). When incorporated into Carbopol 940 hydrogel, Tween 20-based transethosomes achieved 129 μg/cm^2^ cumulative permeation at 24 h, which was 92-fold higher than that of a non-transethosomal gel. Permeation flux (7 μg/cm^2^/h) and permeability coefficient (3.1 × 10^−3^ cm/h) similarly demonstrated order-of-magnitude improvements.

### 3.2. Polymer-Based Nanoparticles

Polymer-based nanoparticles represent a strategically promising approach to enhance the COL ADMET profile. These supramolecular structures (typically 10–500 nm) balance circulation longevity—avoiding rapid renal clearance of smaller particles and mononuclear phagocyte system uptake of larger ones—with tunable physicochemical properties including size, shape, surface charge, and architecture [102]. Their polymeric composition enables programmable drug release kinetics responsive to physiological stimuli (pH, temperature, microenvironmental cues) [103], while facilitating passive accumulation in inflamed tissues via the EPR effect [104,105]. Furthermore, intrinsic polymer affinity to endogenous protein targets (e.g., hyaluronic acid targeting CD44/stabilin-2/TLR4 [106,107]; fucoidan binding P-selectin [108]) or surface functionalization with ligands (e.g., folate for receptor-mediated targeting [109]) enables active tissue-specific delivery [110,111]. This versatility extends across biopolymers (polyamino acids, proteins [112], and polysaccharides [113,114]) and synthetic polymers (polylactic acid (PLA), polyglycolic acid (PGA), polylactide-*co*-glycolide (PLGA), poly-ε-caprolactone (PCL) [115], polymethyl methacrylate (PMMA), polyvinyl alcohol (PVA) [116], PEG [117], etc.), and hybrid systems.

Parashar et al. [118] demonstrated chitosan nanoparticles cross-linked with glutaraldehyde (290–984 nm, PDI < 0.5, +11–14 mV), achieving 93% encapsulation efficiency and 83% drug content. Incorporated into an HPMC hydrogel, they enabled 75% ex vivo rat skin permeation at 24 h and enhanced anti-gout efficacy in a monosodium urate-induced rabbit model. Sadeghzadeh et al. [119] engineered folate receptor-targeted PLGA nanoparticles (250 nm, PDI 0.3, +34 mV) with 90% COL encapsulation efficiency, showing selective cytotoxicity toward HT-29 colon cancer cells via apoptosis induction while sparing HFF normal cells. Zumaya et al. [120] developed PEGylated and non-PEGylated PLGA nanoparticles (~200 nm, PDI 0.1) co-delivering COL and purpurin (70% encapsulation), with release profiles tailored from 4 to 168 h. These exhibited enhanced cellular uptake across Caco-2, PC-3, and MCF cancer lines and synergistic chemo-photodynamic activity.

Hybrid nanoparticles integrate polymers with lipids, proteins, or inorganic components (silica, metal oxides, carbon nanostructures [121]). AbouAitah et al. [122] designed folic acid-conjugated chitosan/cellulose-coated mesoporous silica nanoparticles for COL-curcumin co-delivery. This system demonstrated superior antitumor efficacy versus monotherapies or free drugs, upregulating p53, Bax, and caspase-3 to accelerate apoptosis while reducing cytotoxicity against BJ-1 fibroblasts fourfold compared to free COL.

### 3.3. Colchicine Conjugates

Polymer conjugation addresses nonspecific distribution of COL to off-target organs [123] through two complementary strategies: active targeting via ligand modification [124], and passive targeting exploiting the EPR effect through increased molecular weight [125,126]. However, limited reactive functional groups often necessitate chemical derivatization of COL prior to conjugation [124,127].

Bagnato et al. [124] implemented active targeting by synthesizing a COL-cobalamin bioconjugate through an acid-labile hydrazone linkage. Stability studies confirmed hydrolysis at lysosomal pH (t_1/2_ = 138 min at pH 4.5) but not at physiological pH. In cancer cell models, this conjugate demonstrated 10-fold lower toxicity versus free COL while maintaining nanomolar cytotoxicity comparable to taxanes. Its water solubility highlights its potential for reducing systemic toxicity.

Lagnoux et al. [128] employed glycopeptide dendrimers with cysteine cores for conjugation. COL was deacetylated at C-7 and functionalized for either thioether or disulfide linkages to dendrimers featuring combinatorial hydrophilic/hydrophobic branches. The conjugates exhibited 20–100 times greater selective cytotoxicity toward HeLa cells than toward mouse embryonic fibroblasts, significantly surpassing the 10-fold selectivity differential of free COL.

Addressing conjugation challenges, Svirshchevskaya et al. [129] first synthesized a hydroxyl-bearing furanoallocolchicinoid derivative [127], then succinylated it for carbodiimide-mediated conjugation to chitosan (40 kDa, 94% deacetylation). The resulting amide-linked conjugate disrupted β-tubulin organization in Colo-357 cells (the human pancreatic cancer cell line), inhibiting mitotic spindle formation. In murine Wnt-1 breast tumors, it outperformed free furanoallocolchicine (*p* < 0.05), demonstrating enhanced antitumor efficacy.

### 3.4. Microneedles

Microneedles (MNs) are minimally invasive transdermal devices (less than 1000 μm) that bypass the stratum corneum barrier by creating transient pathways for enhanced drug delivery [130,131]. Fabricated from metals, ceramics, silicon, polymers (e.g., PEG, PLGA, PCL, PS, PVP, and polycarbonate), or hydrogels (e.g., alginate) [132,133,134,135], they are categorized by their functional mechanism into the following groups:

- Solid MNs (“poke and patch”) mechanically perforate the skin before topical drug application [134,136].
- Hollow MNs (“poke and flow”) contain internal cavities for continuous infusion, maintaining patent microchannels for prolonged delivery [137,138,139,140].
- Coated MNs (“coat and poke”) bear drug layers applied via dip-coating or printing; dissolution releases the payload upon insertion, although coating uniformity and loading capacity remain serious limitations [141,142,143].
- Dissolving MNs, the most promising class, incorporate therapeutics within biodegradable polymers (e.g., Soluplus^®^). They enable “poke and release” delivery with high drug loading, sustained release kinetics, and complete biodegradation without residual material [144,145,146].
- Hydrogel MNs combine swelling-controlled release with biocompatibility, though their mechanical properties require optimization [147,148].

Anjani et al. [149] engineered dissolving MNs using COL-loaded Soluplus^®^ (PVP-PCL/PVAc/PEG6000 graft copolymer). The MNs thus synthesized demonstrated robust mechanical integrity and delivered 73% of COL through porcine skin within 24 h. In lipopolysaccharide-stimulated THP-1 macrophages, they suppressed TNF-α production comparably to free COL. Jiang et al. [150] developed hydrogel MNs from disulfide-crosslinked PAM, exhibiting exceptional strength (11.5 N/needle), super-swelling (~2700%), and >95% drug loading. Sustained release (80% over 48 h) and potent in vivo anti-inflammatory activity manifested as reduced production of IL-1β, IL-6, and TNF-α validated their efficacy for suppressing inflammation.

### 3.5. Implants

Cardiovascular interventions—including heart transplantation, coronary artery bypass grafting (CABG), and percutaneous coronary intervention (PCI)—carry significant risks of intimal hyperplasia, defined as pathological migration of vascular smooth muscle cells into the intima, with their subsequent excessive proliferation resulting in narrowing of the vessel lumen (i.e., restenosis) [151,152]. The antiproliferative effect of COL on smooth muscle cells [153] and its capacity to inhibit atherosclerotic plaque formation [154] make it a compelling candidate for preventing arterial restenosis. However, systemic administration, even at low oral doses, induces unacceptable toxicity before therapeutic efficacy is achieved, while localized intramural delivery of COL solutions or microparticles demonstrates limited efficacy [152,155]. These limitations necessitate implantable systems for site-specific drug delivery.

Mishaly et al. [152] pioneered this approach with ethylene vinyl acetate copolymer matrices loaded with COL. Implanted perivascularly in a rat carotid artery balloon injury model, these provided sustained release kinetics (75% over 25 days) and significantly reduced stenosis (11% vs. 39% in controls). Despite promising antirestenotic effects, localized tissue toxicity was observed in some animals, highlighting the need for refined formulations.

Stent-based delivery offers a clinically relevant solution. While the implantation of bare metal stents (BMS) is associated with relatively high restenosis rates, ranging between 16 and 48% [156,157], the use of drug-eluting stents (DES) significantly reduces restenosis due to the presence of polymer coatings that release antiproliferative agents. Paclitaxel-eluting stents initially showed advantages over BMS [158,159], but first-generation durable polymers caused delayed endothelialization and late thrombosis [160]. Contemporary DES employ bioabsorbable polymers (e.g., PLGA) to enhance safety [160,161] and mammalian target of rapamycin (mTOR) inhibitors (sirolimus, everolimus) to suppress neointimal hyperplasia [162,163]. Notably, clinical evidence supports low-dose COL in post-myocardial infarction (MI) patients subjected to PCI [164], with BMS combined with oral COL matching the efficacy of sirolimus/everolimus-eluting DES [163,165]. Synergetic anti-inflammatory and antiproliferative effects of COL motivate the development of COL-eluting stents. Kevina et al. [166] provided the first evidence in favor of this concept using thermosensitive poly(N-isopropylacrylamide-co-N-tert-butylacrylamide) COL-containing films (5 μm thickness, 100 nmol COL/film). Optimized formulations released 0.5–26.0 nmol COL in vitro, significantly inhibiting bovine aortic smooth muscle cell proliferation (≤26%) and migration (≤38%), while maintaining native drug bioactivity, demonstrating potential for COL-releasing DES platforms.

## 4. Colchicine Derivatives

Chemical modification of COL represents a strategic approach to enhance its pharmacological action while reducing inherent toxicity, which persists even at therapeutic concentrations [129]. The structural integrity of Rings A and C is paramount, as they mediate high-affinity tubulin binding and subsequent cytotoxicity [167,168]. Consequently, modifications must not interfere with these pharmacophoric elements.

Permissible alterations are restricted to specific sites: halogenation (Br, Cl, I) at the C-4 position of Ring A, and functionalization (e.g., thiomethyl or amino groups) at C-10 of Ring C [169,170,171]. These targeted adjustments can augment cytotoxic potency and binding affinity. When synergistically combined with more extensive B-ring modifications, they yield derivatives with improved therapeutic indices and selectivity [24,27]. The most efficacious A- and C-ring modifications are detailed below.

### 4.1. A-Ring Modifications

**Figure 4:**
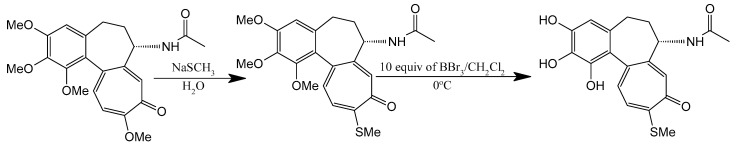
Synthesis of thiocolchicine and 1,2,3-demethylthiocolchicine (adapted from [27]).

**Figure 5:**
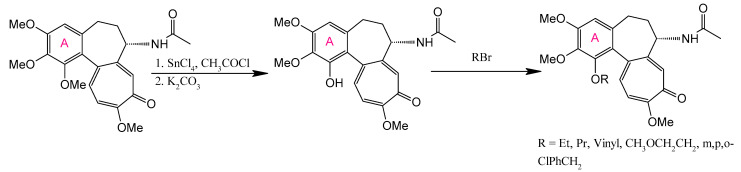
Variation of the C-1 substituent of colchicine (adapted from [27]).

The A-ring of COL presents significant structural constraints for chemical modification, as its 3 methoxy groups (C-1, C-2, C-3) are essential for tubulin binding affinity. Demethylation at any position reduces antimitotic activity by orders of magnitude (Figure 4) [172]. Substitution studies at C-1 with alternative alkoxy or acyloxy groups yielded derivatives with comparable or diminished cytotoxicity, where bulky substituents particularly compromised activity (Figure 5) [173,174]. Consequently, C-4 remains the primary viable modification site that preserves biological function.

**Figure 6:**
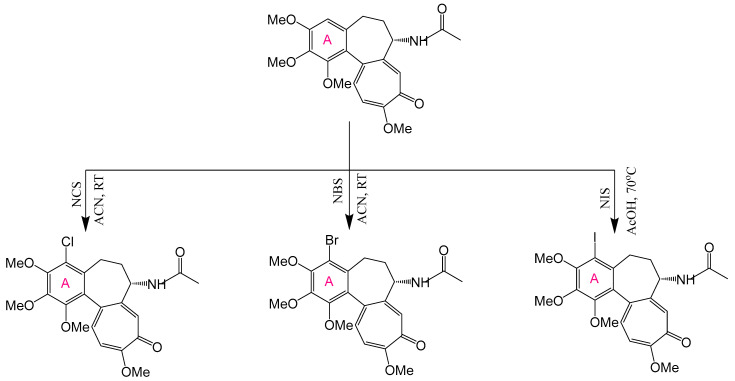
Synthesis of 4-chloro-, 4-bromo-, and 4-iodocolchicine (adapted from [169]).

Halogenation at C-4 significantly enhances anticancer activity, as demonstrated by the synthesis of 4-chloro-, 4-bromo-, and 4-iodocolchicine via electrophilic substitution using N-halosuccinimides under appropriate conditions. The reaction for 4-iodocolchicine was carried out in an acidic medium (acetic acid with heating). Milder conditions were used for 4-chlorocolchicine and 4-bromocolchicine: acetic acid was replaced with acetonitrile, and the reaction occurred at room temperature (Figure 6) [169]. These derivatives exhibited potent activity against A549 (lung), HT-29 (colon), and HCT116 (colorectal) carcinoma cell lines, with particular selectivity toward HT-29 [24]. Alternative electrophilic substitutions at C-4—using formaldehyde in H_2_SO_4_ to yield 4-hydroxymethyl-colchicine, acetic acid for 4-acetoxymethyl-colchicine, or amides for 4-(acylamino)methyl-colchicine [175]—produced less active compounds.

Notably, halogenated derivatives achieved near-complete tubulin polymerization inhibition at low micromolar concentrations, comparable to those of unmodified COL. However, most A-ring modifications generally reduce biological activity and fail to reduce the characteristic toxicity profile of the native molecule, underscoring the structural sensitivity of this pharmacophoric region.

### 4.2. B-Ring Modifications

SAR analyses confirm that the B-ring of COL, while non-essential for tubulin binding and antimitotic activity [167], critically governs biophysical stability and thermodynamic behavior [168,176]. Ray et al. [31] demonstrated that substituent size on this ring inversely correlates with tubulin binding kinetics: smaller groups accelerate association rates while larger moieties slow binding. Furthermore, B-ring modifications significantly influence temperature-dependent binding parameters. These principles have informed the rational design of derivatives where strategic alterations optimize pharmacokinetic properties without compromising target engagement—notably enhancing solubility profiles and metabolic stability while maintaining cytotoxic potency. The size-dependent kinetic effects observed by Ray et al. [31] suggest that steric modulation of the B-ring can fine-tune drug–target residence times, providing a molecular rationale for improved therapeutic indices in next-generation analogs. With this in mind, several COL derivatives have been synthesized and are described below.

#### 4.2.1. Deacetylation at C-7

**Figure 7:**
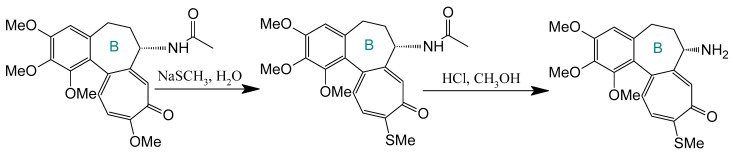
Synthesis of deacetylthiocolchicine from thiocholcine derivative (adapted from [27]).

Deacetylation at the C-7 position generates N-deacetylcolchicine, typically derived from thiocolchicine precursors (Figure 7). Klejborowska et al. [177,178,179] synthesized a series of C-7 amino-functionalized derivatives using this approach. While these compounds exhibited reduced potency against cancer cell lines compared to COL or thiocolchicine precursors, they demonstrated slightly lower cytotoxicity toward normal cells. Critically, the introduced amino group serves as a versatile handle for subsequent functionalization to enhance pharmacological performance.

Sun et al. [180] used deacetylthiocolchicine to create B-nor-colchicinoids via the Demjanov rearrangement, yielding a hydroxymethyl-bearing intermediate. Acylation produced diverse carbonate derivatives, while dehydration with tosyl chloride/1,8-diazabicyclo(5.4.0)undec-7-ene generated a C-7 alkene analog. Evaluated across six cancer cell lines (KB, A549, HCT-8, P388, RPMI-7951, and TE671), the ethoxycarbonate derivative (R = OEt) exhibited cytotoxicity comparable to thiocolchicine. In contrast, the C-7 alkene demonstrated exceptional tubulin polymerization inhibition—surpassing other analogs in mechanistic potency.

Advancing this strategy, Krzywik et al. [181] performed reductive alkylation of N-deacetylcolchicine using aldehydes and sodium cyanoborohydride, creating dual-modified derivatives at C-7 (C-N bond) and C-10 (methylamino group). Remarkably, most compounds exceeded COL, doxorubicin, and cisplatin in cytotoxicity across tested cell lines while overcoming resistance in LoVo/DX cells (a doxorubicin-resistant subline derived from LoVo, a human colon adenocarcinoma cell line), highlighting the therapeutic potential of strategic B-ring functionalization.

#### 4.2.2. Click Chemistry Using Colchicine Azide

**Figure 8:**
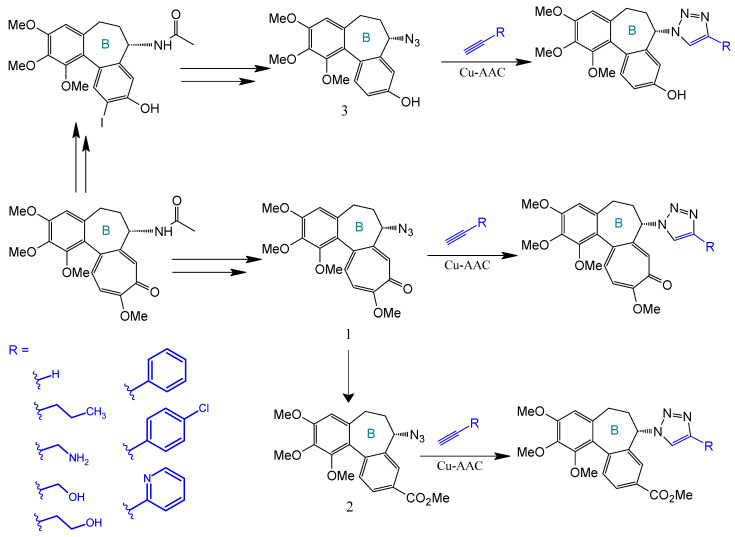
Synthesis of triazoles derived from colchicine, allocolchicine, and N-acetylcolchinol (adapted from [27,187]).

**Figure 9:**
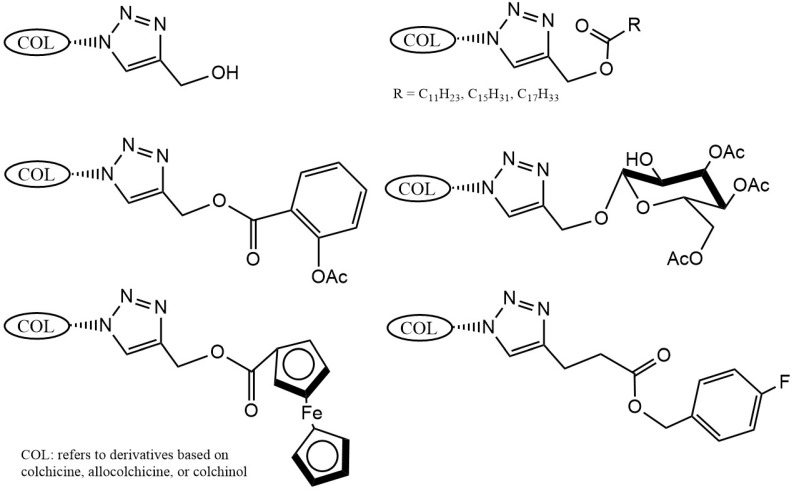
Examples of colchicinoids synthesized by “click” conjugation (adapted from [186,188]).

Strategic derivatization of COL via azide intermediates enables structural diversification through Cu-catalyzed azide–alkyne cycloaddition (CuAAC). As illustrated in Figure 8, three key azide precursors were synthesized: Azide 1 (C7-colchicine azide) via triflyl azide-mediated diazotransfer on deacetylcolchicine [124,182,183]; Azide 2 (allocolchicine azide) through NaOMe/MeOH-mediated rearrangement of Azide 1 [184]; and Azide 3 (colchinol azide) via Windaus oxidation (I_2_/KI/NaOH) followed by reductive deiodination (Zn/AcOH), amide hydrolysis, and triflyl azide functionalization [185]. These azides served as versatile handles for CuAAC reactions with terminal alkynes, generating novel C7-functionalized derivatives of COL, allocolchicine, and colchinol (Figure 9). This methodology enabled the integration of various moieties, including alkyl, aryl, heteroaryl, hydroxyl, amine, ester, carbohydrate, steroid, and organometallic groups, thereby significantly expanding the chemical space of COL [186,187,188,189].

**Figure 10:**
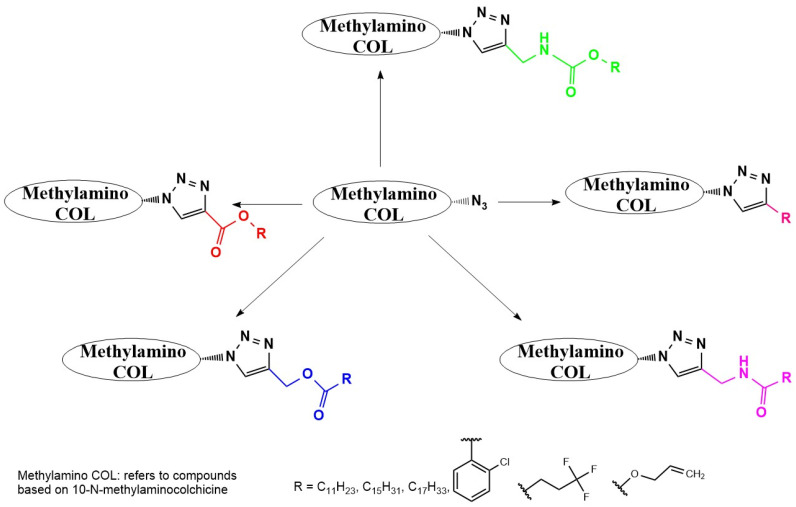
Structural modifications carried out on colchicine to produce 7-triazoles of 10-N-methylaminocolchicine (adapted from [190]).

Krzywik et al. [190] exemplify this approach through a series of 39 triazole derivatives featuring a 10-demethoxy-10-N-methylaminocolchicine core (Figure 10). The strategic replacement of the C10-methoxy group with a methylamino group enhanced cytotoxicity, while triazole C4-substituents spanned ethyl, allyl, pentyl, cyclohexyl, phenyl, carboxyl, carbamate, and amide functionalities. Evaluated against A549 (lung), MCF-7 (breast), LoVo (colon), and drug-resistant LoVo/DX carcinoma cell lines—with BALB/3T3 normal fibroblasts as the control—these compounds demonstrated structure-dependent potency enhancement over native COL, validating click chemistry as a powerful tool for optimizing colchicinoid pharmacophores.

### 4.3. C-Ring Modifications

The tropolone moiety of the C-ring of COL is essential for its high-affinity binding to tubulin [191], yet it exhibits greater synthetic versatility than the A-ring. Numerous C-ring modified colchicinoids retain or enhance biological activity—including derivatives with contracted six-membered rings—while facilitating prodrug development through amine functionalization at C-10 [192].

**Figure 11:**
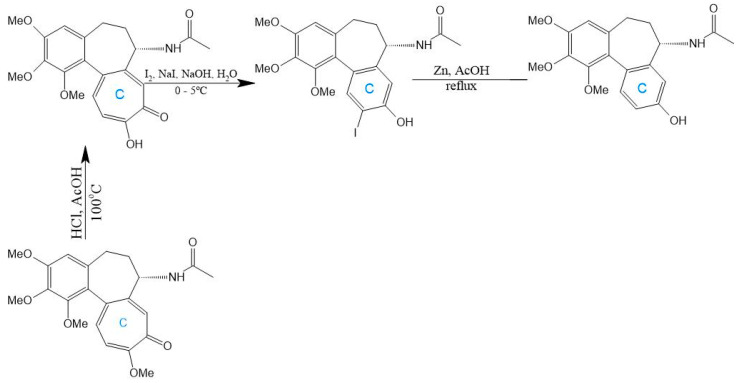
Synthesis of N-acetylcolchinol from colchicine (adapted from [27]).

**Figure 12:**
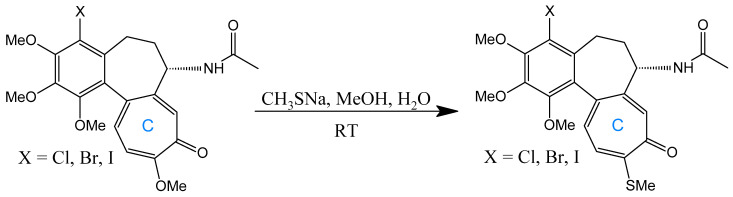
Substitution with thiomethyl groups in C-10 (adapted from [195]).

A seminal C-ring analog, N-acetylcolchinol, is synthesized from natural COL via sequential transformations: acid hydrolysis yields colchiceine, Windaus oxidation (I_2_/KI/NaOH) produces iodocolchinol, and Zn/AcOH-mediated deiodination affords the target compound in high yield (Figure 11) [27,193]. Alternative modifications exploit thiomethyl substitution at C-10, which requires pre-halogenation at C-4 (Figure 12). This stabilization strategy significantly enhances cytotoxicity against A549, MCF-7, LoVo, and ALL-5 cell lines by strengthening tubulin binding [28,194,195].

**Figure 13:**
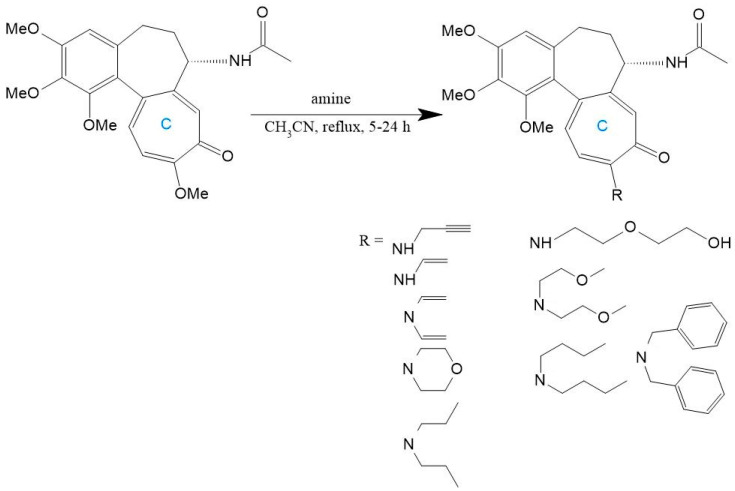
Colchicine derivatives with amino or related functional groups at C-10 (adapted from [196]).

The synthetic malleability of the C-ring is further demonstrated through amino substitutions at C-10. Replacement of the methoxy group with amines generates derivatives that retain bioactivity while evading P-gp-mediated efflux [192]. Huczynski et al. [196] synthesized diverse 10-amino-colchicine analogs via nucleophilic substitution in acetonitrile, incorporating alkylamines, oxygen-containing chains, aromatics, and morpholine rings (Figure 13). Crucially, the amino group at C-10 amplifies anticancer activity while reducing systemic toxicity [197]. Evaluation across HL-60, vincristine-resistant HL-60/vinc, LoVo, doxorubicin-resistant LoVo/DX, and BALB/3T3 cell lines revealed superior potency for derivatives with compact amine substituents, underscoring steric constraints in structure-activity relationships [196].

## 5. Strategic Engineering of Colchicine Delivery Platforms and Derivatives

Optimizing COL’s biopharmaceutical properties requires a strategic nanoformulation approach with two main strategies: (1) combinatorial therapy, which involves co-delivering synergistic agents, and (2) chemical derivatization through prodrug/codrug design. Developing COL-specific delivery systems based on these strategies can significantly improve drug targeting and tissue biodistribution [46,57,198]. Combinatorial therapy addresses the complex pathophysiology of IMIDs by targeting interconnected pathways, enabling dose reduction while minimizing toxicity and resistance development [199]. Clinically, this is exemplified in the management of Behçet’s disease, where COL synergizes with glucocorticoids and anticoagulants to resolve vascular inflammation and thrombosis [200,201]. In these settings, serious adverse effects of systemic glucocorticoids could be attenuated by nanotechnology interventions. For example, our group developed dexamethasone-containing polyelectrolyte complexes and core–shell polymeric nanoparticles, both of which program sustained drug release for a period of hours to months, thereby enhancing therapeutic indices [202,203,204,205,206]. Developing analogous systems for COL-glucocorticoid combinations represents a critical research frontier.

Nanoformulation-mediated co-delivery further enhances combinatorial efficacy through spatiotemporal control [207,208]. High-density lipoprotein (HDL)-reconstituted liposomes exemplify this by combining cholesterol reverse transport with anti-inflammatory drug delivery, simultaneously modulating lipid metabolism and vascular inflammation [209,210,211,212]. Similarly, β-cyclodextrin inclusion complexes have been studied for their cholesterol efflux capabilities while encapsulating therapeutics [213,214]. Additionally, sulfated polysaccharide-based systems, such as chondroitin sulfate, carrageenan, and fucoidan, have been investigated for their antithrombotic, anti-inflammatory, and lipid-lowering properties [215,216,217,218,219,220]. These platforms demonstrate how carrier bioactivity itself becomes therapeutic.

Chemical modification strategies focus on prodrugs and codrugs to widen the therapeutic window of COL [221,222]. Covalent conjugation results in the formation of bioreversible derivatives activated in vivo, such as colchitaxel (COL-paclitaxel glutamate-linked codrug), which releases parent drugs upon hydrolysis [223,224]. Structural hybridization can yield novel pharmacology. For example, adamantane-COL amide conjugates induce unique tubulin clustering in A549 cells, amplifying cytotoxicity through effects depending on the length of the linker [225]. COL-pyronetin ester codrug enhanced VEGF suppression by 1.6-fold in HT-29 cell models by co-targeting telomerase pathways [226].

Organometallic conjugation merges therapeutic and diagnostic capabilities. Gadolinium(III)-DOTA-COL conjugates maintain native pharmacokinetics while enabling T1-weighted MRI visualization in OVCAR-3 xenografts [227], whereas metallocenyl-triazole-COL hybrids exploit reactive oxygen species generation. In particular, ruthenocenyl derivatives exhibit 6–7× increased potency against HepG2 cells, and ferrocenyl analogs double efficacy in HCT116 models [189]. These approaches illustrate how molecular engineering may aid in overcoming traditional ADMET limitations of COL.

## 6. Future Outlook and Challenges

COL represents a promising therapeutic agent for IMIDs and associated comorbidities, particularly atherosclerosis-related cardiovascular pathologies. However, its clinical utility remains constrained by nonspecific biodistribution, a narrow therapeutic window, and dose-limiting toxicity. Advanced delivery strategies are essential to overcome these ADMET limitations.

Nanotechnology-based systems—including lipid-based nanoparticles (SLNs, liposomes, transferosomes, ethosomes), polymeric nanoparticles, and hybrid architectures—offer targeted delivery through passive accumulation at inflammatory sites via the EPR effect. Surface decoration with specific ligands further enables active targeting to affected tissues. These platforms provide precise spatiotemporal control over COL release, enhancing bioavailability while reducing toxicity. Lipid-based systems exhibit particular promise for transdermal administration when incorporated into hydrogels, patches, or microneedles, exploring their stratum corneum penetration capabilities. Localized delivery via polymer-coated vascular stents represents another clinically relevant approach, with COL-eluting stents showing significant potential for preventing post-interventional restenosis. In this particular strategy, the amount of the drug released in the systemic circulation may afford additional benefit in terms of control over the progression of atherosclerosis.

Chemical modification strategies expand the therapeutic arsenal through prodrugs and codrugs. Conjugation with targeting moieties (e.g., cyanocobalamin) or synergistic agents (statins, anticoagulants, glucocorticoids) enhances specificity and efficacy while reducing systemic exposure. Bioactive metal complexes confer additional theranostic functionality, exemplified by gadolinium conjugates enabling simultaneous therapy and MRI visualization. Nanoformulation-mediated co-delivery platforms, such as HDL-mimetic liposomes, cyclodextrin complexes, and fucoidan-based systems, exploit inherent carrier bioactivity to potentiate COL effects through cholesterol modulation, anti-inflammatory action, and vascular protection, respectively.

Despite these advances, translational challenges persist. Scaling nanocarrier production, ensuring batch-to-batch reproducibility, establishing long-term safety profiles, and navigating regulatory pathways represent critical hurdles. Nevertheless, the rational selection of delivery platforms—tailored to specific clinical contexts—holds transformative potential for revitalizing COL therapy. Future research should prioritize scalable manufacturing, predictive preclinical models, and biomarker-guided patient stratification to advance toward personalized IMID management.
